# Observed Touch on a Non-Human Face Is Not Remapped onto the Human Observer's Own Face

**DOI:** 10.1371/journal.pone.0073681

**Published:** 2013-11-08

**Authors:** Brianna Beck, Caterina Bertini, Cristina Scarpazza, Elisabetta Làdavas

**Affiliations:** 1 Centro studi e ricerche in Neuroscienze Cognitive (CNC), University of Bologna, Cesena, Italy; 2 Department of Psychology, University of Bologna, Bologna, Italy; French National Centre for Scientific Research, France

## Abstract

Visual remapping of touch (VRT) is a phenomenon in which seeing a human face being touched enhances detection of tactile stimuli on the observer's own face, especially when the observed face expresses fear. This study tested whether VRT would occur when seeing touch on monkey faces and whether it would be similarly modulated by facial expressions. Human participants detected near-threshold tactile stimulation on their own cheeks while watching fearful, happy, and neutral human or monkey faces being concurrently touched or merely approached by fingers. We predicted minimal VRT for neutral and happy monkey faces but greater VRT for fearful monkey faces. The results with human faces replicated previous findings, demonstrating stronger VRT for fearful expressions than for happy or neutral expressions. However, there was no VRT (i.e. no difference between accuracy in touch and no-touch trials) for any of the monkey faces, regardless of facial expression, suggesting that touch on a non-human face is not remapped onto the somatosensory system of the human observer.

## Introduction

A substantial amount of research has focused on the way that the human brain recognizes emotions from the facial expressions of other humans (see [1] for a review). These expressions serve as communicative signals, conveying information about both the mental state of the other person and the observer's and expresser's shared surroundings. One way in which facial expressions may be recognized is through simulation of the expression in the somatosensory system of the observer, an idea supported by evidence that both actual [1]–[3] and virtual [4] lesions of the somatosensory cortex disrupt recognition of emotional facial expressions. This embodied simulation mechanism would aid emotion recognition by allowing a direct experience of the other's mental state. Furthermore, seeing emotional human faces can enhance tactile perception on the observer's own face, perhaps because such facial expressions are processed in somatosensory cortex and may thus modulate its neural activity [5].

From an evolutionary perspective, the emotional expressions of non-human animals also carry important information for the human observer. They may indicate whether the animal has aggressive or cooperative intentions or signal the presence of potential rewards or threats, such as common food sources or predators. Despite being a source of valuable information for adaptive behavior, few studies have investigated heterospecific facial expression recognition [e.g. 6]–[8] and, to the authors' knowledge, none have examined whether non-human facial expressions are recognized via somatosensory simulation. As with human facial expressions, somatosensory simulation could benefit the observer by providing a direct understanding of the animal's emotional state. One way to investigate this question would be to test whether the sight of touch on emotional, non-human faces modulates tactile perception, which would suggest that non-human facial expressions are likewise processed in the somatosensory system of the human observer.

The interaction between visual face processing and tactile perception can be explored with the visual remapping of touch (VRT) paradigm, wherein viewing touch on a face improves detection of near-threshold tactile stimuli on the cheeks [9]. Electro-tactile stimulation is calibrated to be stronger on one cheek than the other so that extinction of the weaker stimulus occurs approximately half the time that bilateral touch is administered, a pattern that mimics the behavior of patients with damage to the right brain hemisphere who extinguish contralesional stimuli when an ipsilesional stimulus of comparable strength is presented concurrently [10]. In healthy participants, the detection rate of bilateral tactile stimulation increases when seeing a face being touched on both cheeks compared to a non-face object being touched bilaterally and to a face being merely approached by two fingers. Because participants are told that the visual stimuli are non-informative about the task and that they should base their responses solely on what they feel on their own cheeks, this demonstrates an involuntary effect of visual input on a purely tactile task.

VRT is thought to depend upon an established crossmodal effect wherein the sight of touch modulates activity in the observer's somatosensory cortex in the absence of any actual tactile stimulation [11]–[15]. This remapping of seen touch onto the neural system for tactile processing may proceed via feedback signals from multisensory (i.e. visuo-tactile) brain regions to primary (SI) and/or secondary (SII) somatosensory cortex, which may enhance tactile sensitivity on the corresponding body location [13], [14], [16]. Supporting this idea, a functional magnetic resonance imaging study identified a network of fronto-parietal areas involved in VRT that includes the polymodal ventral premotor cortex (VPM) and the face area of SI/SII [17].

To date, VRT studies have only compared human faces to non-face objects, so it is not known whether the effect would extend to non-human faces. Several studies have shown that the human brain processes heterospecific faces differently than conspecific faces after infancy [18]–[24]. Specifically, human faces are analyzed more holistically [18], [20] and more efficiently at an early stage of face processing [19], [23]. Because of these processing differences, and because VRT strength is mediated by perceived similarity to the other [25], one might predict that any VRT for non-human faces would be weaker than for human faces. Nevertheless, VRT might be enhanced if the non-human face expressed fear, a critical emotion for adaptive behavior. Fear recognition is a particularly important function because the fearful expressions of others often signal the presence of an immediate threat in the environment. Efficient recognition of these expressions would allow the observer to quickly enact defensive behaviors to avoid potential harm. Furthermore, the recognition of fearful faces seems especially dependent upon simulation of the facial expression in somatosensory cortex compared to recognizing other emotions from faces [1]–[4]. In keeping with this finding, VRT is enhanced by fearful human faces but not by happy or angry faces [5]. Because fear recognition is important for adaptive behavior and especially dependent upon an embodied somatosensory simulation mechanism, seeing a fearful monkey face being touched might heighten an otherwise weak interspecies VRT effect.

The present study examined whether VRT would occur for monkey faces and whether its strength would be similarly modulated by the monkeys' emotional facial expressions as it is by human facial expressions. Participants reported unilateral or bilateral touch on their own cheeks while they watched fearful, happy, and neutral human or monkey faces being touched or merely approached by fingers. Based on the study by Cardini and colleagues [5], we expected a stronger VRT effect for fearful human faces than for neutral or happy human faces. Furthermore, we hypothesized that VRT would be weak at best for neutral and happy monkey faces but stronger for fearful monkey faces because of the value of fear recognition for survival and the greater representation of fearful expressions in somatosensory cortex [1]–[4].

## Methods

### Ethics statement

This study was approved by of the Ethics Committee for Psychological Research at the Department of Psychology of the University of Bologna. All participants gave written informed consent to participate and were treated in accordance with the ethical standards of the 1964 Declaration of Helsinki.

### Participants

Two separate groups of healthy adult females were recruited. One group (*n* = 12), ranging from 23 to 28 years old (*M* = 25.17 years, *SE* = 0.47), performed a version of the emotional VRT task with monkey faces. The other group (*n* = 14), ranging from 22 to 25 years old (*M* = 23.07 years, *SE* = 0.20), performed the standard emotional VRT task with human faces. All participants had normal or corrected-to-normal vision and reported a normal sense of touch.

### Materials

Four female human faces and four monkey faces showing fearful, happy, and neutral facial expressions were chosen. Human faces were taken from the Pictures of Facial Affect dataset [26]. Monkey faces were gathered from the internet and selected based on emotion categorization and intensity ratings from a separate group of volunteers (Table S1 in Supplementary Materials S1). Short (3000 ms) videos were created in Microsoft Power Point that showed each face on a black background being either touched or approached by one or two human fingers. Care was taken with the monkey videos to ensure that the faces were touched in the less hairy region of skin below the eyes, in case remapping of the seen touch would be hindered by a difficulty in simulating the quality of touch to hairy monkey skin. A computer running C.I.R.O software (http://www.cnc.unibo.psice.unibo/ciro) displayed the visual stimuli and collected responses. Electro-tactile stimulation was delivered via two constant current electrical stimulators (DS7A, Digitimer) connected to two pairs of electrodes (Neuroline, AMBU), one on each side of the participant's face over the zygomatic arch.

### Procedure

Following the staircase procedure used by Cardini and colleagues [5], the detection rate of electro-tactile stimulation was set to nearly 100% on one cheek and to approximately 60% on the other. The cheek that received stronger electro-tactile stimulation (left or right) was counterbalanced between participants. Confirming correct calibration, the mean detection rate of bilateral tactile stimulation across all experimental conditions was 51.74% (*SE* = ±1.18%), and, when bilateral stimulation was not correctly identified, errors mostly consisted of reporting unilateral stimulation on the stronger side (*M* = 95.40% of errors, *SE* = ±1.61%).

**Figure 1:**
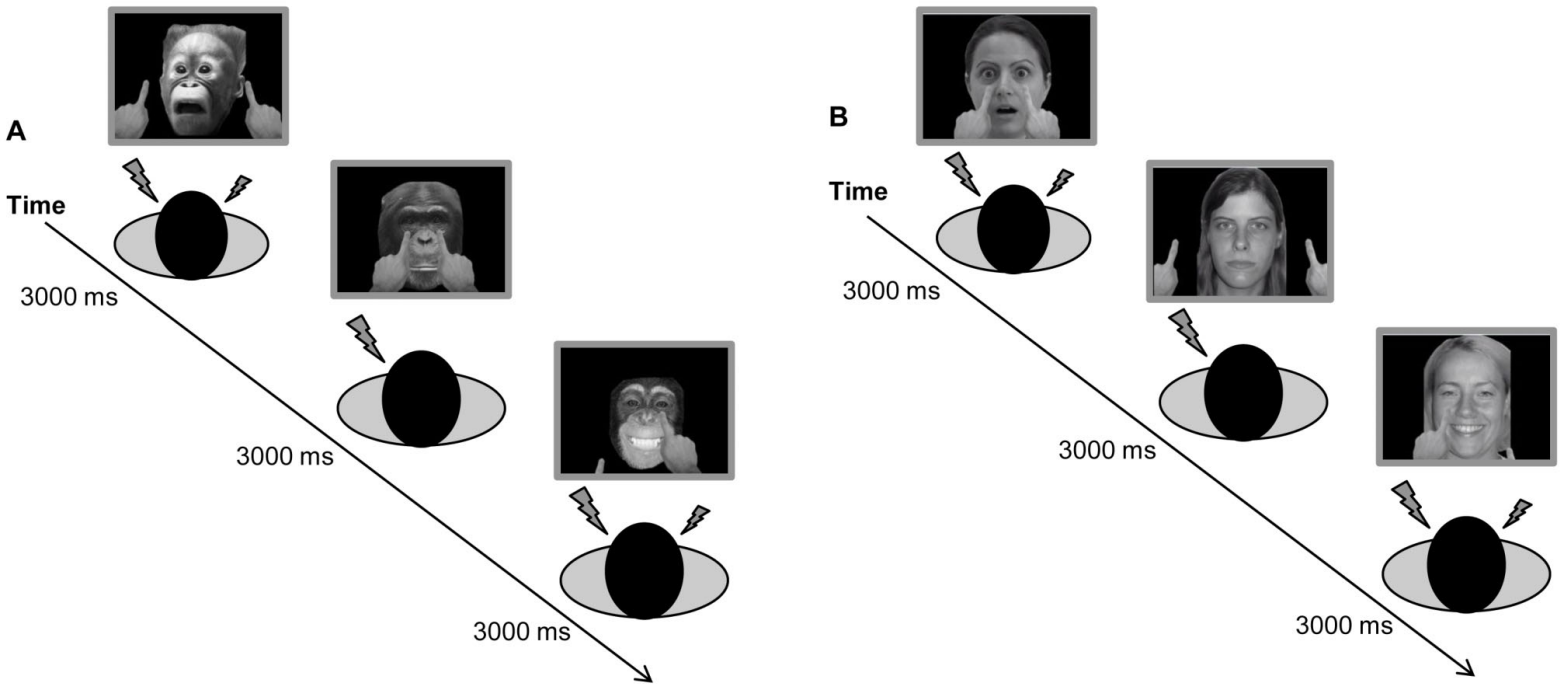
Schematic depiction of experimental trials showing example stimuli. Sample trials from the group that saw monkey faces (A) and the group that saw human faces (B) are shown, each one combining one of two types of tactile stimulation (unilateral or bilateral), one of two types of visual stimulation (unilateral or bilateral), and one of two types of finger trajectory (touch or no-touch). Please note that each block contained only one type of facial expression. Fearful, neutral, and happy expressions are shown together in this figure for illustrative purposes only. Human faces were taken from the Pictures of Facial Affect dataset [26]. (Note that the human faces shown here are similar but not identical to the actual face stimuli used, and are thus for illustrative purposes only. The actors gave their written consent to have their likenesses published.) Monkey faces were gathered from the internet and rated by a separate group of volunteers for emotion category and intensity (Table S1 in Supplementary Materials S1).

The experiment consisted of three blocks of VRT trials, one with neutral faces, one with fearful faces, and one with happy faces. Block order was counterbalanced between participants, and electro-tactile detection thresholds were re-calibrated between blocks. Each trial began with a face in the center of the screen and two fingers at the bottom of the screen on either side of the chin. One or both of the fingers then moved upward and either touched the cheek on the same side of the screen or touched a location about 5 cm lateral to the face before returning to the bottom of the screen. When the fingers reached the top of their trajectory (approximately 1000 ms into the trial), electro-tactile stimulation was delivered to one or both of the participant's cheeks (Figure 1). Participants used a keyboard to indicate whether they felt touch on the left cheek (the “D” key), on the right cheek (the “K” key), or on both cheeks (the space bar). They were instructed to respond as quickly and accurately as possible, and informed that the location of apparent touch on the cheeks of the other face was non-informative about the touch on their own face. Each trial combined one of two types of tactile stimulation (unilateral or bilateral), one of two types of visual stimulation (unilateral or bilateral), and one of two types of finger trajectories (touch or no-touch), resulting in 8 trial types that were repeated 12 times in each block for a total of 96 trials per block, presented in a random order. Only trials with both bilateral tactile stimulation and bilateral finger movement (touch or no-touch) were analyzed.

## Results

**Figure 2:**
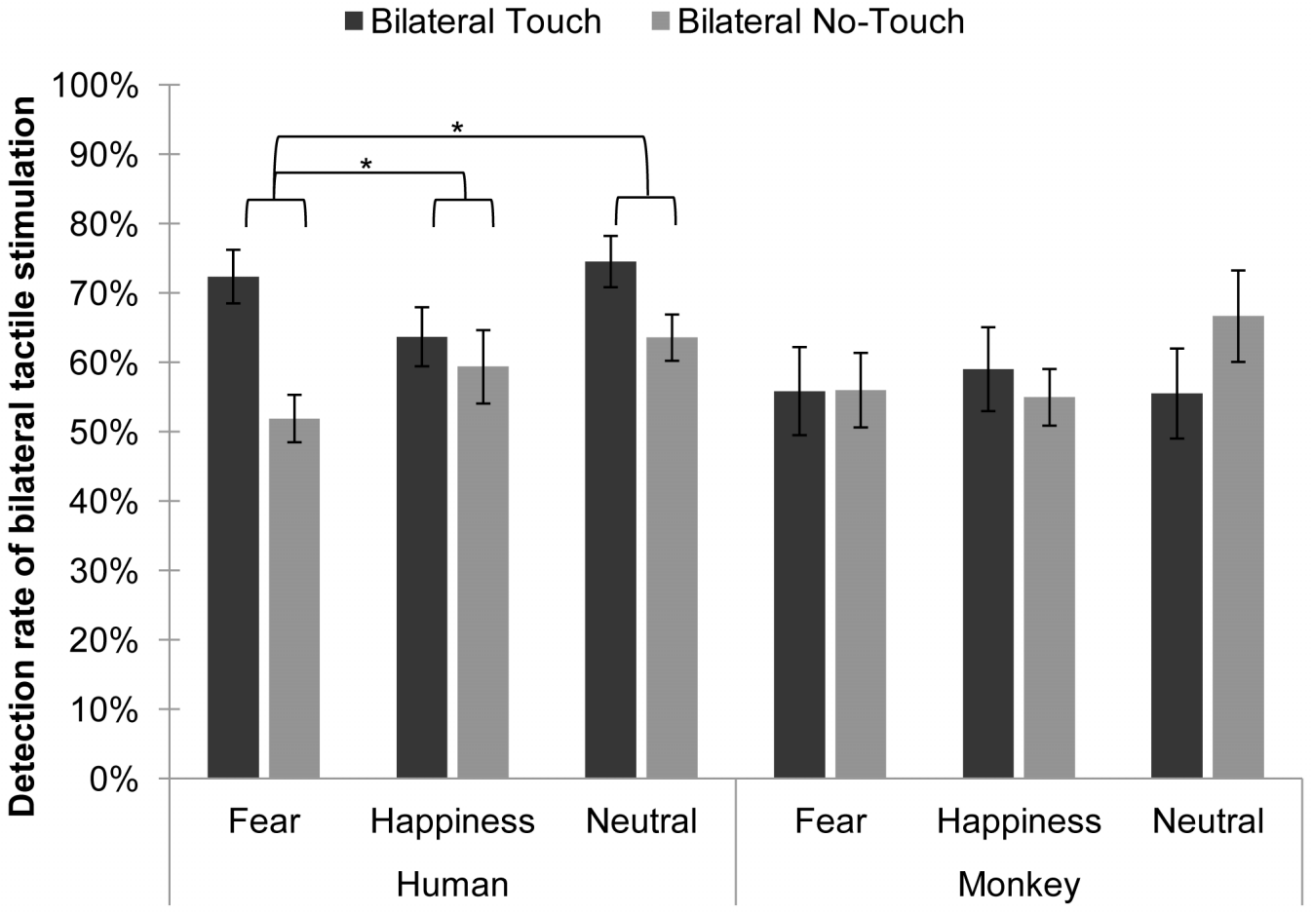
Mean (±*SE*) detection rates of bilateral tactile stimulation in each condition. Asterisks indicate significant (*p*<.050) differences in the magnitude of VRT (bilateral detection rate in the touch condition minus bilateral detection rate in the no-touch condition) between emotional expression conditions in the group that saw human faces. No such comparisons were made in the group that saw monkey faces because the 3 (facial expression) ×2 (finger trajectory) ANOVA was not significant.

An analysis of variance (ANOVA) with the between-subjects factor of species (human or monkey) and within-subjects factors of facial expression (fearful, happy, or neutral) and finger trajectory (touch or no-touch) was conducted on the percentages of correct bilateral responses in each condition. None of the main effects were significant, but there was a two-way interaction between finger trajectory and species, *F*(1, 24) = 9.01, *p* = .006, η_p_^2^ = .27, and a three-way interaction between facial expression, finger trajectory, and species, *F*(2, 48) = 3.21, *p* = .049, η_p_^2^ = .12. To elucidate these interactions, two separate 3 (facial expression) ×2 (finger trajectory) within-subjects ANOVAs were conducted, one on the data from the group that saw monkey faces and another on the data from the group that saw human faces. The monkey face group did not show any main effects, nor was the interaction significant (*p*≥.277 in all cases), indicating that VRT did not occur in any of the monkey expression conditions (Figure 2). In the human face group, there was a main effect of finger trajectory, *F*(1, 13) = 30.72, *p*<.001, η_p_^2^ = .70, with higher accuracy in bilateral touch trials (*M* = 70.20%, *SE* = ±2.71%) than in bilateral no-touch trials (*M* = 58.28%, *SE* = ±3.05%). There was also an interaction between facial expression and finger trajectory, *F*(2, 26) = 9.94, *p* = .001, η_p_^2^ = .43. T-tests comparing the VRT effect (the bilateral detection rate in the touch condition minus the bilateral detection rate in the no-touch condition) in the three human expression conditions (Fear Touch – No-Touch: *M* = 20.48%, *SE* = ±3.68%; Happy Touch – No-Touch: *M* = 4.33%, *SE* = ±2.34%; Neutral Touch – No-Touch: *M* = 10.96%, *SE* = ±2.85%) showed that VRT was greater for fearful faces than for happy faces, *t*(13) = 4.13, *p* = .001, and neutral faces, *t*(13) = 2.57, *p* = .023 (Figure 2). The VRT effect for neutral faces also seemed to be greater than for happy faces, though this difference did not reach two-tailed significance, *t*(13) = −2.02, *p* = .064.

## Discussion

Corroborating previous studies, seeing a human face being touched improved detection of near-threshold tactile stimuli simultaneously delivered to the observer's own face [9], and this effect was enhanced by fearful facial expressions compared to neutral or happy ones [5]. This is consistent with fear recognition being particularly dependent upon embodied simulation in somatosensory cortex [1]–[4]. A simulation mechanism of emotion recognition might be especially valuable for recognizing emotions such as fear that indicate an immediate threat to survival, as the direct experience of the emotion might allow an observer to quickly identify and react to the threat.

Because the human brain processes human and non-human faces differently [18]–[24] and the VRT effect is mediated by perceived similarity to the other [25], we predicted that any VRT effect for monkey faces overall would be minimal. This hypothesis was borne out. While the standard increase in bilateral tactile perception for touch trials was observed with human faces, the group that saw monkey faces did not exhibit this enhancement, suggesting that only observed touch on the faces of conspecifics is remapped onto the observer's own somatosensory cortex at a level capable of enhancing tactile perception on the face. Though some previous studies have found that the sight of touch on inanimate objects triggers SII activity [12], [13], seeing touch on body parts also modulates SI activity [11], [14], [15] and enhances SII activation beyond that found when viewing non-body objects being touched [11]. Either or both of these differences might account for VRT being specific to viewing touch on a body part. The present study further suggests that the remapping of seen touch onto somatosensory cortex in a manner that enhances tactile perception is not only specific to seeing touch on a body, but to seeing touch on a human body.

Contrary to expectations, showing monkey faces with fearful expressions did not increase tactile perception on touch trials compared to no-touch trials. Perhaps fearful monkey faces do not enhance VRT because there is no initial VRT effect for monkey faces to modulate, and the presence of a fearful expression in itself is not enough to influence tactile perception on the face. It is also possible that the fearful expressions of monkeys, unlike those of humans, are not processed via simulation in the observer's somatosensory system. To the authors' knowledge, no studies have investigated whether viewing monkey facial expressions modulates activity in human somatosensory cortex. Viewing non-emotional monkey face actions (biting and lip smacking) activates human mirror neuron systems in the inferior parietal lobule and the inferior frontal gyrus, which respond to both the execution and observation of actions [27]. Nevertheless, viewing fearful monkey faces, unlike fearful human faces, does not enhance amygdala activity compared to neutral (chewing) faces [28]. As the amygdala is involved in both expressing fear [29]–[31] and recognizing fear in others [31]–[33], this could be taken as evidence that monkey facial expressions are not simulated in the same way as human facial expressions. Future studies should investigate whether recognizing the emotional facial expressions of non-human primates involves processing in somatosensory cortex, as does recognition of human facial expressions [1]–[4].

Note that, for the present study, the important factor was not the significance of the monkey's emotional expression to other monkeys but to the human participants. The monkey faces in the fearful and happy conditions were selected because they were consistently identified as fearful expressions or happy expressions in the pilot study (Table S1 in Supplementary Materials S1), which, like the main experiment, used volunteers who were novices in reading the emotional expressions of non-human primates. An interesting follow-up to this study would be to test people who work with monkeys and would therefore have more experience with identifying their emotional expressions. There is evidence that expertise with a species can improve recognition of their emotional expressions [34] and change the way that their social body signals are processed in the brain [35]. Future studies could examine whether such expertise could result in embodied simulation of the emotional expressions of non-human animals. Specifically, one could investigate whether experts in the social signals of a non-human species (e.g. animal trainers) remap observed touch on those animals onto their own somatosensory systems, and whether this potential VRT effect is modulated by the animal's emotional expression. In the case that experts do show a VRT effect for touch on the non-human animals they are familiar with, this would indicate that VRT is not restricted to conspecifics per se but to members of species with which one has interacted extensively, learning their nonverbal social cues. Furthermore, if this potential VRT effect were mediated by the emotional content of the facial or bodily expression, this would suggest that the embodied simulation mechanism of emotion recognition is also experience-dependent rather than strictly limited to conspecifics.

### Conclusion

Seeing a human face being touched enhances detection of concurrent near-threshold tactile stimulation on the observer's own face, and this visual remapping of touch (VRT) is heightened if the observed face expresses fear [5]. The present study demonstrated that VRT only occurs when seeing touch on human faces. Seeing a monkey's face being touched did not improve tactile perception compared to seeing the same face not being touched, indicating that observed touch on non-human faces is not simulated within the human observer's somatosensory system. Furthermore, seeing a monkey face with a fearful expression being touched did not induce VRT, suggesting either that human observers do not simulate the emotional expressions of non-human animals in their own somatosensory systems or that the simulation of a fearful expression in itself is not enough to modulate tactile perception on the face.

## Supporting Information

Supplementary Materials S1 **A description of the pilot study procedure and analysis used to select the monkey facial expression stimuli.** Table S1, Emotion categorization and intensity ratings for monkey facial expressions.
(DOCX)
