# Supplementary material for: Observed Touch on a Non-Human Face Is Not Remapped onto the Human Observer's Own Face
Source: PLoS One. 2013 Nov 8;8(11):e73681. doi: 10.1371/journal.pone.0073681 (PMC3826747; doi:10.1371/journal.pone.0073681)
Supplement: Supplementary Materials S1 — A description of the pilot study procedure and analysis used to select the monkey facial expression stimuli. Table S1, Emotion categorization and intensity ratings for monkey facial expressions. (DOCX) [file pone.0073681.s001.docx]

**Supplementary Materials S1**

A group of 32 volunteers categorized 26 monkey facial expressions as either happy, sad, angry, fearful, or neutral, and rated the intensity of each emotional expression on a scale from 1 (“not at all intense”) to 6 (“very intense”). Emotion categorization and intensity data for the 12 monkey expressions selected for the main experiment are shown in Table S1. To analyze the emotion categorization data, scores of 1 and 0 were assigned to instances of correct and incorrect categorization, respectively, and the mean score for each participant in each expression category (fearful, happy, and neutral) was calculated. These mean scores were then entered into a Friedman’s ANOVA for non-parametric data, which showed a significant difference between accuracy of emotion categorization in the three categories, *X^2^*(2) = 34.81, *p* < .001. Post-hoc Wilcoxon signed-rank tests showed that neutral facial expressions were correctly categorized less often than fearful (*p* < .001) or happy (*p* < .001) expressions, which did not differ from each other in emotion categorization accuracy (*p* = .750). Because participants were engaged in an emotion categorization task, they may have looked for subtle emotional signals in neutral faces that they would not otherwise have attended to. Importantly, neutral faces were still identified as neutral 71.88% of the time, and they were not consistently miscategorized as a certain emotion. Among the instances of neutral face miscategorizations, 52.78% were sadness misidentifications, 33.33% were happiness misidentifications, and 13.89% were anger misidentifications.

Shapiro-Wilk tests indicated that intensity ratings were not normally distributed, so they were also analyzed with a Friedman’s ANOVA. Intensity ratings differed significantly among the facial expression categories, *X^2^*(2) = 38.58, *p* < .001. Wilcoxon signed-rank tests revealed that neutral expressions were perceived to be less intense than fearful (*p* < .001) and happy (*p* < .001) expressions, and happy expressions were also perceived to be less intense than fearful expressions (*p* = .010). It is appropriate that the neutral faces should be perceived to be less intense than either type of emotional expression. Though the fearful and happy faces were not equivalent in terms of emotion intensity, this does not impede interpretation of the results. Both were perceived to be quite intense, yet neither resulted in VRT, suggesting that the intensity of the monkey’s emotional expression was not a relevant factor.

Table S1

*Emotion Categorization and Intensity Ratings for Monkey Facial Expressions*

| Facial Expression | Face Identity | Categorization Accuracy | Mean Intensity Rating |
| --- | --- | --- | --- |
| Fear | Face 1 | 100.00% | 5.03 |
|  | Face 2 | 100.00% | 4.44 |
|  | Face 3 | 96.88% | 4.91 |
|  | Face 4 | 96.88% | 5.25 |
|  | Mean | 98.44% | 4.91 |
| Happiness | Face 1 | 100.00% | 4.84 |
|  | Face 2 | 93.75% | 4.66 |
|  | Face 3 | 96.88% | 4.34 |
|  | Face 4 | 96.88% | 4.38 |
|  | Mean | 96.88% | 4.55 |
| Neutral | Face 1 | 75.00% | 3.21 |
|  | Face 2 | 75.00% | 3.73 |
|  | Face 3 | 65.63% | 3.17 |
|  | Face 4 | 71.88% | 4.14 |
|  | Mean | 71.88% | 3.56 |

*Note*. Participants in the pilot study categorized each facial expression as happy, sad, angry, fearful, or neutral, and then rated the intensity of each expression on a scale from 1 (“not at all intense”) to 6 (“very intense”).
